# Natural Deep Eutectic Solvents (NADES) as a Tool for Bioavailability Improvement: Pharmacokinetics of Rutin Dissolved in Proline/Glycine after Oral Administration in Rats: Possible Application in Nutraceuticals

**DOI:** 10.3390/molecules21111531

**Published:** 2016-11-14

**Authors:** Marta Faggian, Stefania Sut, Beatrice Perissutti, Valeria Baldan, Iztok Grabnar, Stefano Dall’Acqua

**Affiliations:** 1 Department of Pharmaceutical and Pharmacological Sciences, University of Padova, Via Marzolo 5, 35131 Padova, Italy; marta.faggian@studenti.unipd.it (M.F.); stefania_sut@hotmail.it (S.S.); valeria.baldan.4@studenti.unipd.it (V.B.); 2 Department of Chemical and Pharmaceutical Sciences, University of Trieste, P.le Europa 1, 34127 Trieste, Italy; bperissutti@units.it; 3 Faculty of Pharmacy, University of Ljubljana, Askerceva cesta 7, SI-1000 Ljubljana, Slovenia; Iztok.Grabnar@ffa.uni-lj.si

**Keywords:** NADES, HPLC-MS, rutin, pharmacokinetics

## Abstract

There is a need for innovation in plant-derived pharmaceuticals, food supplements and nutraceutical products regarding the use of more eco-sustainable solvents for their extraction. Furthermore, the poor oral bioavailability of several phytochemicals with health promoting effects stimulates the research in the field of pharmaceutical formulations. Natural Deep Eutectic Solvents (NADES) are formed by natural compounds, and can be considered as future solvents being especially useful for the preparation of nutraceuticals and food-grade extracts. In this paper various NADES were prepared using sugars, aminoacids and organic acids. Rutin (quercetin-3-*O*-α-l-rhamnopyranosyl-(1→6))-β-d-glucopyranose) was used as a model compound to study NADES. Moreover, the effect of various eutectic mixtures on rutin’s water solubility was studied. Proline/glutamic acid (2:1) and proline/choline chloride (1:1) mixtures have a solubility comparable to ethanol. The proline/glutamic acid (2:1) eutectic containing rutin was used in a pharmacokinetic study in Balb/c mice while bioavailability was compared to oral dosing of water suspension. Plasmatic levels of rutin were measured by HPLC-MS/MS showing increased levels and longer period of rutin permanence in plasma of NADES treated animals. This paper reports the possible use of non-toxic NADES for pharmaceutical and nutraceutical preparations.

## 1. Introduction

Health promoting products such as herbal medicines, food supplements or nutraceuticals obtained by solvent extraction from plants or foods are widespread. However, the conventional extraction methods used have several drawbacks, namely low selectivity and residual solvent in the final products. Due to their safety level, the most commonly used solvents for nutraceutical production are water, ethanol or aqueous ethanol mixtures. Unfortunately, these mixtures may be scarcely efficient in extraction due to the variable nature and polarity of extractable bioactive compounds [1,2,3,4]. Additionally, many claimed active ingredients of nutraceuticals have poor bioavailability. Thus the need for further research in the field of nutraceutical and pharmaceutical formulation to enhance the oral absorption of such compounds. A new approach in this area may be represented by the so called deep eutectic solvents (DES) or Low Transition Melting Mixtures (LTMM). These solvents are mixtures of organic compounds having significantly lower melting points than their individual components. They have been developed as an alternative to other solvents, namely, the ionic liquids (IL), i.e., salts possessing particular physicochemical properties (viscosity, density, hydrophilicity, solubility), which may be tuned by combining different cations and anions [5]. IL are not allowed in food or food supplement production due to their potential toxicity and are generally avoided due to their “non-natural” origin. In recent years, some special DES that were produced using natural products have been studied and generally called Natural Deep Eutectic Solvents. These new solvents have gained much attention from the scientific community, especially in the green chemistry area, for they have a potential for replacing common organic solvents presenting inherent toxicity and high volatility, thereby releasing volatile organic compounds in the atmosphere [6,7,8]. In this context, Natural Deep Eutectic Solvents (NADES) comprising natural compounds, such as organic acids, amino acids and sugars, have been put forward. NADES are obtained by the complexation of a hydrogen acceptor and a hydrogen-bond donor. Such solvents are almost non-volatile at ambient condition, are chemically and thermally stable, non-flammable, and have good solvent properties for several organic compounds [5,7,9].

While the poor solubility of several bioactive compounds in water and ethanol mixtures is a severe limitation in the extraction of food supplements and nutraceutical bioactive ingredients [3,10,11,12,13], any alternative ideal solvent should present high level of safety and eco-sustainability as well as improved extraction performances. The DES capacity for the extraction of bioactive natural products is correlated with their physical–chemical properties, including H-bonding interactions, polarity, viscosity, and pH. The high extractability of phenolic acids with DES may be attributed to H-bonding interactions between DES molecules and phenolic compounds. The polarity of DESs is an important factor affecting their extraction efficiency. Nevertheless, NADES can be considered also as “ingredients” in a nutraceutical or functional food, and offer the possibility of combining various molecules, leading to the preparation of tailor-made solvents for solutes. The combination of various molecules in different NADES leads to the preparation of tailor-made solvents designed to extract solutes with different properties (polarity, charge etc.) [4,6,7,8,14,15]. Radošević at al. studied various phenolic grape skin extracts obtained by using NADESs and tested their biological activity in two human tumor cell lines (HeLa and MCF-7). Results show that NADES components could be chosen not only to fine-tune solvent physicochemical characteristics but also to enhance biological activity of extracts prepared in NADESs [16]. Due to the food grade property of these ingredients, it is assumed that extracts obtained by NADESs may be directly used in products for human consumption without the need for expensive downstream purification steps [17,18]. This was demonstrated by in vitro cytotoxicity assays using two human cell lines (MCF-7 and HeLa) of a few tailor made NADES. The tested NADESs possessed low cytotoxicity, which makes them good candidates for the green extraction, leading to the novel application of NADES in food and pharmaceutical industry [16].

DES have also been taken into consideration for pharmaceutical applications. Morrison et al. considered them as solvents for low water soluble drugs, including griseofulvin, itraconazole and danazol [19,20]. Recently deep eutectic solvent derivatives (DESD) were used for the solubilization of poorly water-soluble drugs such as itraconazole, piroxicam, lidocaine, and posaconazole while the enhanced drug solubility and the DESD properties were considered attractive for topical formulations of such drugs [21].

The use of NADES as extraction solvents appears a promising approach in the field of nutraceuticals, especially for natural products with poor bioavailability. To our knowledge, the literature in this field is very scarce. In the present paper, NADES were investigated as solubilizers using rutin as a model compound. Rutin (3′,4′,5,7-tetrahydroxy-flavone-3-rutinoside) is a common dietary glycosylated flavonoid present in fruits, vegetables and in many plant-derived beverages such as tea and wine [22]. It has been extensively studied due to its anti-inflammatory [23], antibacterial [24], cancer chemopreventive [25] and antidepressive [26] activities. Furthermore, it is used in pharmaceutical and nutraceutical products as a phlebotonic drug, although literature supporting these effects is still limited [27]. Rutin has been widely used for the treatment of chronic venous insufficiency; further uses that have been proposed are glaucoma, hay fever, haemorrhoids, varicose veins, poor circulation, oral herpes, cirrhosis, stress, low serum calcium, and cataract [28]. Pharmacokinetics and oral absorption of rutin and aglycone quercetin were previously studied in healthy volunteers at various dose levels [19]. Quercetin glucuronides and/or sulfates were measured in plasma, and no rutin was detectable, indicating the intense metabolism of these compounds. The authors claimed that prior to absorption rutin is hydrolized. Additionally, there are two pharmacokinetic studies with oral administration of rutin in rats. Recently, rutin and quercetin as patented polyherbal formulations were co-administered in rats by gavage, and bioavailability was compared to that of the co-administration of the two pure compounds at equivalent doses [29]. The observed differences indicated that from the polyherbal formulation bioavailability of rutin had increased, while bioavailability of quercetin has decreased when compared to the co-administration [29]. In a methodological paper describing an HPLC-MS/MS method for rutin quantification in rat plasma, preliminary pharmacokinetic data were published showing the maximum concentration (1659 ng/mL) 5 min after 2.5 mg/kg sublingual vein administration of rutin [28]. He et al. reported that the three flavonoid glycosides (rutin, astragalin and isoquercitin) were rapidly absorbed and eliminated from rat plasma after oral administration of total flavonoids from mulberry leaves [30].

The aim of this study was to evaluate the use of food-components NADES as vehicles for rutin administration and to estimate their influence on rutin bioavailability. Thus a series of NADES were prepared using various compounds. Rutin solubility was evaluated in various eutectic systems comparing the results with water and ethanol. In order to study the possibility of using NADES as administration vehicles, the proline/glutamic acid (2:1) eutectic was tested in a pharmacokinetic study in Balb/c mice using 10 mg/kg oral dose of rutin, and compared to the same dose of rutin suspended in water.

## 2. Results

### 2.1. Rutin Solubility in the Different Prepared NADES

**Figure 1:**
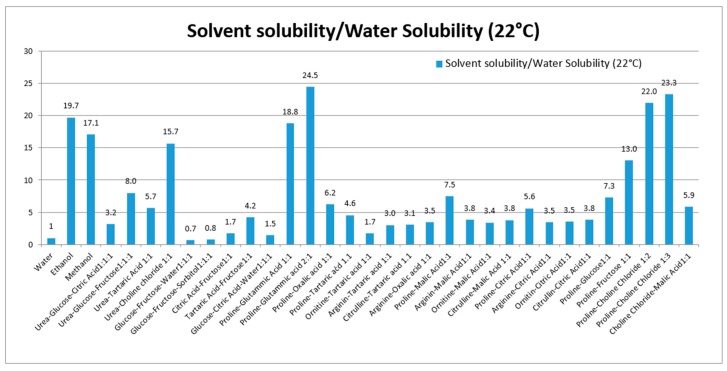
Solubility ratio of rutin (solvent/water) in each prepared NADES. The solubility in all solvents were significantly different from solubility in water (*p*-values < 0.05).

Various NADES composed of urea, amino acids, sugars and choline were used to solubilize rutin. Thirty NADES were selected and used for evaluation of rutin solubility. Water and ethanol were used as reference solvents due to their favorable use as solvents in the production of extracts for nutraceuticals and functional foods. Water was selected as a reference, because of the poor rutin solubility (120 μg/mL at 22 °C). For comparison purposes, the solubility ratio (solvent/water) was used. The solubility and solubility ratios of rutin in each solvent are reported in Table 1 and summarized in Figure 1.

These eutectics, prepared using different starting materials, can be divided into five groups, the first is based on urea (1–4), the second on polyalcohols and sugars (5–6), the third on organic acid and sugars (7–9), the fourth on organic acid and amino acid (10–27), and the fifth group was prepared using choline chloride, sugars and amino acid (28–30). Our results show the ability of various NADES to dissolve rutin. To our knowledge, no published data are available related to the in vivo effects of NADES as administration tools for bioactive constituents.

### 2.2. HPLC-MS/MS Method Validation

#### 2.2.1. Specificity, linearity, LOQ and LOD

**Figure 2:**
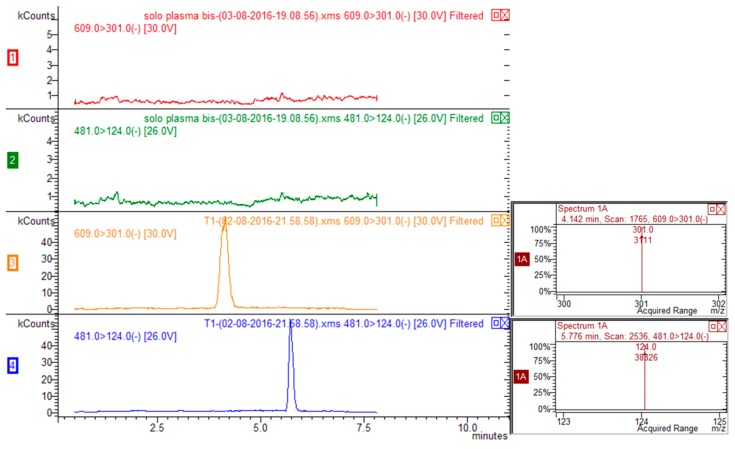
HPLC-MS/MS chromatograms corresponding to transitions 609 > 301 for rutin and 481 > 124 for ISTD (silimarin) of blank plasma (traces red and green for rutin and ISTD respectively) and plasma spiked with rutin and ISTD (traces yellow and blue for rutin and ISTD respectively).

Exemplary multiple reaction monitoring (MRM) chromatograms for rutin-spiked plasma (rutin 20 ng/mL, ISTD 100 ng/mL) are reported in Figure 2. Five calibration mixtures prepared mixing different ratios of rutin/ISTD (namely, 0.053, 0.0106, 0.0181, 0.0363, 0.0727, 0.1453) were used to prepare calibration curve (y = area of rutin/area of ISTD; x = quantity of rutin/quantity of ISTD) that was linear and reliable over the considered calibration range. Limit of quantification (LOQ) was 3 ng/mL and limit of detection was 1 ng/mL for rutin.

#### 2.2.2. Accuracy and Precision

Spiked samples were assayed for intra-day and inter-day precision and accuracy at concentrations of 10, 20, 80 ng/mL. Data are summarized in Table 2.

### 2.3. Pharmacokinetics in Mice of Proline-Glutamic Acid Rutin Eutectic and Rutin Water Suspensions

**Figure 3:**
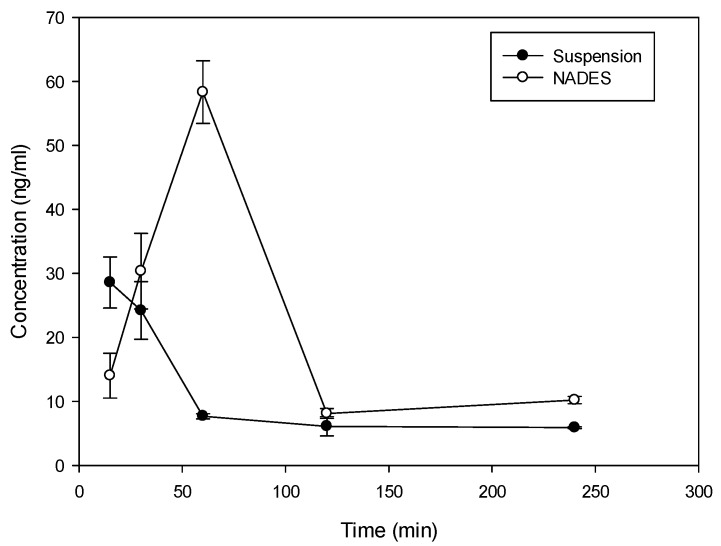
Time course (mean ± SD) of plasma concentration time course of rutin in Balb/c mice following oral administration of 10 mg as proline/glutamic NADES or suspension in water.

Considering the solubility properties nine NADES (2, 3, 4, 10, 11, 17, 28, 29, 30) were able to solubilize half of the rutin compared to the best solvent (ethanol). On the other hand, eutectic 11 and 28 (proline/glutamic acid 2:1 and proline/choline chloride 1:2 respectively) resulted to be the most promising NADES presenting rutin solubilization higher than ethanol and methanol. These results showed the ability of different NADES to dissolve rutin. Mixture 11 (Proline–Glutamic Acid 2:1) was selected to this purpose, because it contains only two amino acids and is therefore suitable for oral administration. Selected rutin NADES 11 formulation (Proline–glutamic acid: 2:1) and rutin as a water suspension were administered orally to mice, and plasma levels were determined up to 4 h after administration. As reported in Figure 3 and Table 3, different plasma levels were observed.

Rutin (10 mg dose dissolved in 0.5 mL of solvent) in NADES 11 and as water suspension was administered orally to mice and the plasma levels were determined up to 4 h after administration. As demonstrated in Figure 3 and Table 3, incorporation of rutin in NADES markedly affected rutin plasma levels.

With both formulations, absorption of rutin was fast with t_max_ at 15 min following administration of suspension and 60 min following the administration of NADES (Figure 3). The elimination from plasma was also rapid with a terminal half-life of 106 min and 86 min for suspension and NADES, respectively. Despite the difference in t_max_, MRT of rutin with the two formulations was similar (158 min vs. 131 min) indicating that there is no remarkable difference in the absorption rate. There was, however, a large difference in C_max_ and AUC. Relative bioavailability of NADES vs. suspension was 2, indicating approximately 100% improvement in the extent of rutin absorption with NADES, presumably due to improved solubility. Thus, the use of NADES 11 can be considered as an interesting tool for solubilization and administration of rutin due to valuable pharmacokinetic properties.

## 3. Discussion

Recently Dai et al. investigated the possibility to modulate NADES properties as solvents for poorly soluble natural compounds, also by adding small amount of water decreasing viscosity and improving conductibility [8]. For example, a previously published paper considered the mixtures sucrose-choline chloride, lactic acid–glucose and proline–malic acid; these NADES were reported to be efficient for polyphenol extraction from *Cartamus tinctorius* L. [18]. The solubility and physical properties of NADES can be modulated by adding water to the composition. The supermolecular complex structures of proline choline NADES are preserved when the content of water is below 50% while further dilution produces a solution of the free forms of the individual components in water, showing that gradual changes in the structure of NADES during dilution may affect their physicochemical properties and also their applications [8]. Recently, Aroso et al. proposed therapeutic deep eutectic systems prepared using choline chloride or menthol conjugated with three different active principles, namely, acetylsalicylic acid, benzoic acid and phenylacetic acid. Their results indicated the potential of these eutectics as dissolution enhancers in the development of novel and more effective drug delivery systems. However, their experiments were limited to in vitro trials only [31].

## 4. Experimental Section

### 4.1. Chemicals

NADES have been prepared using mixtures of sugars (glucose, fructose), amino acids (glutamic acid, proline, arginine, citrulline, ornithine), organic acids (citric acid, malic acid, oxalic acid, tartaric acid), and other compounds containing nitrogen (urea and choline chloride). As regards sugars and polyols, glucose were purchased from Carlo Erba (Milan, Italy), fructose, from Sigma-Aldrich (Milan, Italy). As regards organic acids, citric and oxalic acids were purchased from Riedel-De-Haen AG (Seelze, Germany), tartaric acid from Codex (Turin, Italy), malic acid from Carlo Erba. As regards amino acids and derivative, alanine was purchased from Merck (Vimodrone (MI), Italy), histidine from Sigma-Aldrich, proline, arginine, citrulline and ornithine from Fagron (Bologna, Italy). Choline chloride was purchased from Sigma-Aldrich and urea from Alfa Aesar (Karlsruhe, Germany). Rutin and internal standard silimarin were purchased from Sigma-Aldrich. Solvents as acetonitrile and methanol of HPLC grade were purchased from Scharlab (Riozzo di Cerro al Lambo (MI), Italy), formic acid from Carlo Erba reagents.

### 4.2. NADES Preparation

For NADES preparation we used the previously reported approaches described in the review of Dai et al. 2013 [6].

### 4.3. Solubility Trials and Quantification of Solubilized Rutin in the NADES by HPLC-DAD

Exactly weighed quantity of rutin was suspended in water, ethanol, methanol and in the various prepared NADES with a concentration of 2.5 mg/mL. Samples were stirred on a magnetic stirrer (Stuart, Bibby Scientific Ltd., Stone, Staffordshire, UK) for one hour at room temperature and centrifuged for 21 min at 13,000 rpm with Eppendorf 5415 R centrifuge The time of one-hour stirring was chosen because it was sufficient for all the prepared mixtures to reach maximum concentration of rutin. For quantitative measurement of solubilized rutin, a portion (100 μL) of the clear supernatant obtained after centrifugation was diluted 1:5 mL in DMSO. Dilution is necessary to assess the amount of rutin because of the high concentration of solutions and because of the high viscosity of NADES.

For quantification standard solution of rutin (100 μg/mL) was prepared dissolving rutin in methanol with an ultrasound bath. Calibration curve was obtained injecting standard solution at different concentrations namely (50, 25, 10, 5 and 1 μg/mL) Calibration curve was as follows y = 17.832 x + 9.7822 (R^2^ was 0.9998). Limit of Quantification was 1 μg/mL.

For HPLC-DAD a series 1260 HPLC instrument (Agilent, Cernusco Sul Naviglio (MI), Italy) equipped with a quaternary pump, a diode-array detector, an auto sampler and a column oven compartment was used. Analyses were performed on Eclipse XDB C_8_ column (5 μm, 4.6 mm × 150 mm, Agilent). The mobile phase was (A) water-formic acid (99.9:0.1, *v*/*v*) and (B) acetonitrile. A gradient program was used as follows: [0 → 10th min: A:B (90:10) → A:B (60:40) 10 → 11th min: A:B (60:40) → A:B (0:100) 11 → 12th min: A:B (0:100) → A:B (0:100) 12 → 13th min: A:B (0:100) → A:B (90:10) 13 → 14th min: A:B (90:10) → A:B (90:10)]. The mobile phase flow rate was 1.2 mL/min and the injection volume was 10 μL. The chromatogram was recorded at 350 nm and spectral data for all peaks were obtained in the range of 190–400 nm. The retention time of rutin in the analysis conditions was 3.8 min.

### 4.4. Animals Blood Collection and Extraction

All experimental protocols involving animals were reviewed and approved by the Ethical Committee for animal Experiments of the University of Padua (CEASA; 49571). Female, Balb/c mice (8–10 weeks old) were housed (three per cage) in polycarbonate cages and kept on a 12 h light/dark cycle. Food and water were given ad libitum. Mice, randomly divided into two groups of 15 animals each, received 10 mg of rutin (20 mg/mL) by oral gavage as water suspension or proline glutamic acid 2:1 NADES. A single blood sample was collected by cardiac puncture from each animal at 15, 30, 60, 120, or 240 min after dosing. Whole blood samples were heparinized. Three samples were obtained per each time point and each treatment group.

### 4.5. HPLC-MS Plasma Analysis

Standard stock solutions for determination of rutin in mice blood were prepared by dissolving rutin and internal standard silimarin in methanol (100 μg/mL and 150 μg/mL respectively).

Rutin stock solution was diluted 1:100 (100 μg/mL → 1 μg/mL) and silimarin (ISTD) stock solution was diluted 1:10 (150 μg/mL → 15 μg/mL). The calibration curve was obtained mixing 100 μL of 15 μg/mL IS with different volume (400, 200, 100, 50, 25, 10, 5 and 2.5 μL) of 1 μg/mL rutin standard solution in order to obtain different rutin/silimarin quantity ratios. Mixture of IS and rutin were added to blank plasma samples and used for sample and calibration curve preparation. To 400 μL of whole blood 100 μL of IS solution were added (161 ng) and acidified methanol was added (300 μL) in order to precipitate proteins. Sample was vortexed and subjected to five minute ultrasound bath at room temperature. The sample was then centrifuged and the clear supernatant was transferred to an Eppendorf tube and dried under nitrogen flow. 200 μL of methanol were then used to redissolve the solid and used for HPLC-MS/MS measurements.

For measurement an Agilent series 1260 HPLC chromatograph equipped with a Prostar 410 autosampler (Varian, Cernusco Sul Naviglio (MI), Italy) and coupled with Varian 320 TQD MS spectrometer was used. The mass spectrometer was equipped with electrospray ionization (ESI) source as the interface and analysis was conducted in negative ion mode for both the analytes. Analyses were performed on a Pursuit XRs 3 C_18_ column (50 mm × 2.0 mm, Varian). The mobile phase was (A) water-formic acid (99:1 *v*/*v*) and (B) acetonitrile. A gradient program was used as follows: [0 → 1th min: A:B (85:15) → A:B (85:15) 1 → 7th min: A:B (85:15) → A:B (0:100) 7 → 8th min: A:B (0:100) → A:B (85:15) 8→11th min: A:B (85:15) → A:B (85:15)]. The mobile phase flow rate was 220 μL/min. The injection volume was 10 μL.

The ESI source was set in negative ionization mode. Quantification was performed using multiple reaction monitoring (MRM) with *m*/*z* 609 → 301 transition for rutin and *m*/*z* 481 → 124 transition for IS. The MS parameters were capillary voltage −115 V, needle voltage −4400 V, shield voltage 600 V, collision energy 26 V, Q1 voltage 0.5 V and Q3 voltage 2.6 V, nebulising gas pressure 50 psi and drying gas pressure 25 psi. Calibration curve using the ratio Peak area Rutin/Peak area IS versus quantity rutin/quantity IS was y = 2.8515 x + 0.0002. The Limit of Detection was 0.3 ng/mL and the Limit of Quantification was 1.2 ng/mL.

### 4.6. Method Validation

Assay specificity was evaluated comparing the chromatograms of standard-spiked plasma with blank plasma from three different sources. Calibration curves were fitted by least square regression analysis to plot peak area ratio of rutin/ISTD relatively to the ratio of the amount of rutin/ISTD. Limit of Quantification (LOQ) was calculated as the lowest amount with a relative standard deviation < 20%. Intra and inter day stability, extraction recovery, matrix effects were measured. Precision and accuracy were evaluated using QC samples (*n = 5*) at concentrations of 10, 20 40 and 80 ng/mL on three different days. Different plasma samples were used for intra- and inter-day stability, extraction recovery, and matrix effects with five replicates.

### 4.7. Pharmacokinetic Analysis

Non-compartmental pharmacokinetic analysis was performed using WinNonlin Version 2.1 (Pharsight Corporation, Mountain View, CA, USA) software. The area under the mean plasma concentration-time curve extrapolated to infinity (AUC) was calculated using the linear trapezoidal method. Maximum concentration and the time when it was observed (C_max_ and t_max_, respectively) were reported as observed. Terminal half-life (t_1/2_) was calculated as t_1/2_ = ln2/λ_z_, where λ_z_ is the slope of the terminal phase of the plasma concentration-time curve in the semi-log plot calculated by linear regression. Mean residence time (MRT) was calculated as AUC/AUMC, where AUMC is the area under the first moment curve calculated by linear trapezoidal method. Relative bioavailability of NADES versus suspension was estimated as a ratio of AUC following the administration of NADES and AUC following the administration of the suspension.

## 5. Conclusions

In this study we showed the possibility of producing amino acid-based NADES with good capability of dissolving the polyphenol rutin. The most promising mixture was proline/glutamic acid (2:1) being able to dissolve a comparable amount of rutin as ethanol and twenty times higher than water. The compounds selected for NADES preparation in this paper are available in a normal diet and can be administered orally at moderate doses without major health hazards. Administration of rutin equidoses in mice, as water suspension or as solution in NADES, resulted in different pharmacokinetic profiles. Rutin absorption was fast in both cases, yet four times slower than in water suspension. The elimination from plasma was also rapid, but with a longer terminal half-life of NADES. For this reason, proline/glutamic acid (2:1) NADES may improve bioavailability due to the increase of rutin solubility. The obtained in vivo data indicate that the oral administration of rutin with proline/glutamic acid (2:1) NADES improve bioavailability of this polyphenol compared to the water suspension. This effect may be related to the fact that the NADES formulation allow the administration of rutin as a solution being more available for the absorption by the gastrointestinal tract. This preliminary study showed the potential of NADES as solubilizing and formulating agents for polyphenols administration. Further studies are needed to deeply understand the role of different NADES in the bioavailability of poorly soluble natural products.

## Figures and Tables

**Table 1:** Solubility of rutin (mean ± SD of 3 determinations) in each solvent. The solubility in all solvents were significantly different from solubility in water (*p*-values < 0.05).

Solvent Class	Mixture Number	Solvent	Rutin Solubility at 22 °C (μg/mL)
Reference solvent		Water	120.0 ± 4.9
		Ethanol	2369.7 ± 93.2
		Methanol	2053.7 ± 89.7
Urea based	1	Urea–Glucose–Citric Acid 1:1:1	378.7 ± 8.5
	2	Urea–Glucose–Fructose 1:1:1	961.3 ± 30.6
	3	Urea–Tartaric Acid 1:1	679.8 ± 19.0
	4	Urea–Choline chloride 1:1	1883.3 ± 48.1
Sugar based	5	Glucose–Fructose–Water 1:1:1	81.9 ± 2.5
	6	Glucose–Fructose–Sorbitol 1:1:1	90.8 ± 1.9
Sugar and organic acid based	7	Citric Acid–Fructose 1:1	205.1 ± 5.1
	8	Tartaric Acid–Fructose 1:1	504 ± 16.4
	9	Glucose–Citric Acid–Water 1:1:1	175.2 ± 3.7
Organic acid and amino acids based	10	Proline–Glutamic Acid 1:1	2255.9 ± 63.4
	11	Proline–Glutamic Acid 2:1	2938.4 ± 117.9
	12	Proline–Oxalic Acid 1:1	749.3 ± 22.5
	13	Proline–Tartaric Acid 1.1	546.9 ± 19,9
	14	Ornitine–Tartaric Acid 1:1	209.7 ± 5.7
	15	Arginine–Tartaric Acid 1:1	362.7 ± 11.2
	16	Citrulline–Tartaric Acid 1.1	370.4 ± 13.1
	17	Arginine–Oxalic Acid 1:1	414.3 ± 13.6
	18	Proline–Malic Acid 1:1	900.3 ± 31.1
	19	Arginin–Malic Acid 1:1	457.4 ± 17.8
	20	Ornitine–Malic Acid 1:1	408.0 ± 14.8
	21	Citrulline–Malic Acid 1:1	454.5 ± 18.5
	22	Proline–Citric Acid 1:1	672.5 ± 26.2
	23	Arginine–Citric Acid 1:1	414.3 ± 13.3
	24	Ornitine–Citric Acid1:1	424.7 ± 17.2
	25	Citrulline–Citric Acid 1:1	459.7 ± 14.4
	26	Proline–Glucose1:1	878.7 ± 31.3
	27	Proline–Fructose 1:1	1563.9 ± 43.8
Choline chloride based	28	Proline–Choline Chloride 1:2	2642.8 ± 101.3
	29	Proline–Choline Chloride 1:3	2799.2 ± 103.3
	30	Choline Chloride–Malic Acid 1:1	702.0 ± 22.3

**Table 2:** Intra-day and inter-day precision and accuracy at different concentrations.

	Nominal Concentration (ng/mL)	Measured (ng/mL)	RSD (%) *	Accuracy (%)
Intra day (*n* = 5)	10	10.76 ± 0.30	3.2	107.5
	20	19.64 ± 0.79	4.9	98.2
	40	38.87 ± 1.10	2.8	97.1
	80	80.46 ± 1.88	2.4	100.5
Inter day (*n* = 5)	10	10.22 ± 0.40	3.9	102.2
	20	19.44 ± 0.61	3.2	97.2
	40	39.11 ± 1.86	4.7	97.8
	80	78.79 ± 1.80	2.9	98.4

* Relative Standard Deviation.

**Table 3:** Non-compartmental pharmacokinetic parameters of rutin following oral administration in Balb/c mice (dose 10 mg).

Pharmakokinetic Parameter	Suspension	NADES
t_max_ (min)	15	60
C_max_ (ng/mL)	28.6	58.3
t_1/2_ λ_z_ (min)	106	86
AUC_last_ (ng min/mL)	2225	4862
AUC (ng min/mL)	2888	5806
AUC (%Extrapolated)	23	16
MRT (min)	158	131
